# The Social Insurance Literacy Questionnaire (SILQ): Development and Psychometric Evaluation

**DOI:** 10.1007/s10926-023-10159-7

**Published:** 2023-12-30

**Authors:** Christian Ståhl, Elin Karlsson, Marika Wenemark, Jan Sandqvist, Kristofer Årestedt

**Affiliations:** 1 Division of Education and Sociology, Department of Behavioural Sciences and Learning, Linköping University, Linköping, Sweden; 2 Division of Society and Health, Department of Health, Medicine and Caring Sciences, Linköping University, Linköping, Sweden; 3 Unit for Public Health and Statistics, East Region, Linköping, Sweden; 4 Division of Prevention, Department of Health, Medicine and Caring Sciences, Rehabilitation and Community Medicine, Linköping University, Linköping, Sweden; 5 Faculty of Health and Life Sciences, Linnaeus University, Kalmar, Sweden; 6 Department of Research, Kalmar, Kalmar County Sweden

**Keywords:** Sick leave, Social insurance, Instrument development, Methodological study, Psychometrics, Rasch measurement theory

## Abstract

**Purpose:**

For clients to understand social insurance decisions and processes, information from authorities needs to be comprehensible, and clients need sufficient individual abilities. These dimensions are captured by the concept *social insurance literacy*, which has been operationalized into a measure, the Social Insurance Literacy Questionnaire (SILQ). The aim of this study was to describe the development of the SILQ and evaluate its psychometric properties using Rasch measurement theory.

**Methods:**

The development of the SILQ included a Delphi study and cognitive interviews. A preliminary version, divided on four scales corresponding to the domains of the concept (obtaining information, understanding information, acting on information, and system comprehensibility) was psychometrically evaluated according to Rasch measurement theory, in a survey to a stratified random sample of people on sick leave (*n* = 1151) sent out in the fall of 2020.

**Results:**

Overall, the items in the final version of the SILQ demonstrated good fit to the Rasch model, and the response scale worked as intended. Unidimensionality was supported for all scales, but minor problems with local dependency was detected for three items. The person separation was 0.80 for the Obtain scale, 0.82 for the Understand scale, 0.68 for the Act scale, and 0.81 for the System scale. Corresponding ordinal alpha values were 0.91, 0.91, 0.86, and 0.91, respectively.

**Conclusion:**

This study is a first step toward exploring literacy in the social insurance field. The SILQ covers individual abilities and systems’ comprehensibility, and the results show that it has acceptable psychometric properties.

**Supplementary Information:**

The online version contains supplementary material available at 10.1007/s10926-023-10159-7.

## Introduction

Although social insurance systems are aimed at offering financial support to people who are unable to provide for themselves, e.g., due to sickness, this aid is not necessarily given in an equal and fair manner. Several studies have shown differences between groups of clients on sick leave, regarding whose eligibility is questioned and who receives interventions [1–4], and other studies state that communication characteristics may affect the outcome in terms of receiving sickness benefits and to which extent clients perceive that the outcome and procedures are just [5]. Further, the quality of information is essential for clients to understand and accept procedures and decisions [6]. In this context, the concept of literacy may be useful to explain some of the differences in outcomes and perceptions. In previous research, literacy has been explored and conceptualized in different contexts, such as health literacy, financial literacy, health insurance literacy, legal capability and social security literacy, aimed at explaining both individuals’ understanding of a specific context or phenomena and the comprehensibility of said context, and how these factors affect various outcomes [7].

To explore interactions between individuals and social insurance systems and how this relates to the social resources of individuals and the communication strategies of systems, the concept *social insurance literacy* was developed through a scoping review and in dialogue with an international expert panel [7]. The aim of developing the concept was to arrive at a useful way of making sense of the individual and contextual aspects that may affect peoples’ experiences of interacting with a complex insurance system, by identifying and operationalizing different domains that may influence such interactions. An underlying assumption is that the level of social insurance literacy will have an effect on how well individuals succeed in claiming benefits, and that a person with strong individual abilities may be better served by the system, especially if the system is complex and the information is hard to understand. Another is that a system may be better or worse equipped to meet people with different individual abilities, which means that the system side of the concept is equally important for how interactions will play out. The concept may therefore offer an alternative to other concepts such as social capital (which is more explicitly focused on social resources and access to networks), and administrative literacy (which is focusing on individual comprehension of documents and regulations) [8]; social insurance literacy both aims to provide a more context-specific terminology, and to combine individual and contextual aspects.

In the scoping review, the concept was defined as “the extent to which individuals can obtain, understand and act on information in a social insurance system, related to the comprehensibility of the information provided by the system” ([7], p. 1783). The definition is primarily based on the literature on health literacy, for which there exists numerous definitions and measures. This literature has developed into more dynamic and contextually influenced definitions, and we specifically wanted to include domains to reflect such complexity in our definition. Hence, we identified four domains, of which three were individual (obtaining, understanding, and acting upon information, specifically focusing on information required for claiming sickness benefits), and one contextual (system comprehensibility, which refers to the individual’s experience of how well the insurance agency manages to provide information that is possible to understand). Based on this definition, we proceeded to develop the Social Insurance Literacy Questionnaire (SILQ) with the aim to operationalize these four domains into a measure which could be used for assessing both individual abilities required for claiming benefits in an adequate way, and how well the communication of insurance agencies work, i.e., whether the information delivered is actually considered comprehensible. The SILQ explores areas of social insurance literacy that are of both practical and scientific interest in relation to the workings of sickness insurance systems, and how well public officials provide accessible, understandable, and transparent information [7]. Such information may be used by agencies to improve their communication, as well as by researchers to study inequalities in how well systems treat people with different abilities and backgrounds, i.e., a discriminative purpose, or an evaluative purpose to study changes over time [9].

To receive a reliable and valid measure and to make accurate decisions about social insurance literacy, there is a need for a rigorous psychometric evaluation of the SILQ. Overall, there are two dominating psychometric paradigms, classical test theory (CTT) and item response theory (IRT) [10, 11]. In modern psychometric literature, IRT models are often favored since they override important limitations of CTT, for example that CTT is test and sample dependent, and that reliability and standard error of measurement are assumed to be constant for all persons taking the test [12]. The Rasch model is commonly categorized as an IRT model but has also been described as an own theory, the Rasch Measurement Theory (RMT) [11]. The main difference between IRT and RMT is that RMT specifies uniform item discrimination, while IRT allow un-uniform item discrimination parameters (2-parameter models) and guessing parameters (3-parameter models) [12]. Consequently, IRT typically describes data while RMT obtains data that fits the model. Therefore, RMT is the most restrictive of the three paradigms and commonly described as the most appropriate for evaluations of patient-centered outcome measurements [13]. The Rasch model is a mathematical probabilistic model, meaning that it predicts the probability of a person’s response to an item, given their latent trait level and the difficulty of the item. The model relies on two key assumptions, unidimensionality and local independence. Unidimensionality implies that the latent trait being measured is the only important factor that influences a person’s response to an item and local independence implies that the residual correlations should not be correlated; after controlling for the latent trait, the responses to different items should be independent of each other [10].

### Objective

The aim of this study was to describe the development of the SILQ and to evaluate its psychometric properties using Rasch measurement theory.

### The Swedish Sickness Insurance Context

Swedish sickness insurance is a universal system available for everyone who lives or works in Sweden. The Swedish Social Insurance Agency (SIA) is responsible for assessing clients’ eligibility for sickness benefits and coordinating vocational rehabilitation. Eligibility exists for up to 90 days if the client is unable to do their regular work, and up to 180 days if the client cannot do any other work provided by the employer. From day 181, eligibility criteria are stricter as the assessment is carried out in relation to any potential work in the regular labor market. Initially, a sick note from the clients’ treating physician is enough to receive sickness benefits, but the SIA can choose to issue a standardized work ability evaluation when further information upon the clients’ pre-requisites is required. This evaluation is performed by specific units within health care, and involves a physician and, if necessary, an occupational therapist, physiotherapist, and/or psychologist. The officials at the SIA make the final assessment of clients’ work ability and eligibility for benefits. For this assessment, officials are not allowed to take individual or social factors such as age, educational level or labor market situation into consideration.

In work ability assessments, social insurance literacy is likely to have importance both on an individual level, i.e., the person’s knowledge about regulations and ability to understand and act on such knowledge, and on an organizational level, i.e., how well the officials communicate and explain regulations and procedures. For the assessment after day 180, this is likely to be of increased importance since the assessment is stricter.

## Methods

Development of a new assessment instrument is often conducted step by step in a number of ordered phases to make sure that it accurately measures the constructs of interest [14, 15]. In summary, the development process contains three phases: *planning*, *construction*, and *evaluation* [14]. Each phase consists of several steps that should be taken to satisfy the requirements for completing that phase [15].

The phase of *planning* begins with a formulation of the purpose of the instrument (discriminative, predictive, evaluative), what the instrument is designed to measure (construct), and definition of the target group for the instrument (population) [14]. This phase also contains a review to ensure that no similar valid and reliable instrument already exist on the market for assessment of the construct(s) of interest, which for the present case was reported in a previous study [7]. Modern instrument development also emphasizes the importance of using both experts and representatives from the targeted respondent group to ensure content validity from different perspectives [16]. The *construction* phase begins with developing a table of specifications in order to narrow the purpose and to form the content areas (domains) of the instrument. It includes constructing operational definitions of assessment items and a rating scale for the new instrument, as well as revising and reducing the number of items. It also includes an initial evaluation of the instrument’s face validity. The *evaluation* phase focuses on testing the instrument’s qualities and psychometric properties. To be able to claim with acceptable certainty that an instrument is valid or reliable, it is necessary to examine several different forms of validity and reliability [17, 18].

### Study Procedures

#### Planning and Construction Phase

Based on the conceptualization of social insurance literacy, CS, EK, and JS developed a first draft of the SILQ in English covering the four domains of the definition, i.e., system comprehensibility, and the individual ability to obtain, understand, and act upon information. During fall 2018 an international expert panel (*n* = 7) with expertise within working life research and/or sickness insurance systems (representing sociology, law, insurance medicine, occupational medicine, and social medicine) was recruited based on their area of expertise and experience. This panel evaluated the SILQ through a Delphi-study [19]. The Delphi rounds were qualitative and consisted of a Word-document of the emerging draft of the SILQ which the participants were invited to comment on. The draft was also presented and discussed in a workshop at the European Public Health conference in Ljubljana in November 2018. Comments were focused on the definition and the conceptual framing of the project, e.g., if it should focus on a specific social insurance system or be more general, where the latter was chosen. Other comments focused on overlap between domains, and revisions, inclusion, and exclusion of items in the questionnaire, e.g., adding appeals to decisions. Through this process, content validity was evaluated, i.e., whether the content reflects the scope that was intended [20], i.e., whether the questionnaire corresponded to the content of the definition as well as the contextual relevance in different jurisdictions. The SILQ was revised in between rounds by CS, EK, and JS. Consensus was reached after three rounds.

The initial version of the SILQ was translated from English to Swedish by CS, EK, and JS for a validation study in a Swedish context in a population of people with experience from sick leave in the Swedish sickness insurance system. This version was translated back to English by a native English-speaking colleague to ensure the quality of the translation, leading to a few minor clarifications. Thereafter a statistician specialized at questionnaire design (MW) was consulted which led to restructuring and condensing of the questionnaire. Examples of changes at this stage was changing the wording of items to direct questions and removing items that were too similar.

The next step in the development phase involved cognitive interviews with five clients who had experience of contacts with the sickness insurance system, to evaluate if respondents were able to understand and answer the questions [16]. These participants were recruited through the researchers’ personal networks. First, three cognitive interviews were performed by CS and EK, and after a minor revision of wording, two more interviews with new respondents were conducted to form a preliminary version of the SILQ for a psychometric evaluation, which included 47 items covering the four domains. The Obtain domain included 7 items, the Understand domain 12 items, the Act domain 11 items, and the System domain 17 items. Respondents to the SILQ were asked to evaluate their ability and the system’s comprehensibility on each item on a four-point response scale: ‘Very good’ (score of 0), ‘Rather good’ (score of 1), ‘Rather bad’ (score of 2), and ‘Very bad’ (score of 3), and a N/A option. This means that a high score indicates worse literacy.

### Evaluation Phase

To evaluate the psychometric properties of the SILQ, an invitation to participate in a survey was sent by mail to 3993 clients in Sweden who were on sick leave (180–720 days duration of the current spell) in August 2020. To facilitate analyses also of clients who had their sickness benefits withdrawn, the selection was stratified to include 1173 clients whose applications for benefits were denied after the 180-day assessment, with the rest having ongoing cases. The stratified random sample was drawn by the SIA, who also provided the researchers with postal addresses. The invitation included information about the study, that participation was voluntary, and that the data would be treated with confidentiality. The first invitation included a web address to an online questionnaire with a personal login. Respondents had the opportunity to log in an unlimited number of times to continue completing the questionnaire but could only submit their answers once. A first reminder was sent by mail in October and included an information letter along with a paper version of the SILQ questionnaire. A second reminder was sent as a postcard in late November 2020. The web survey closed on December 31st, 2020, but paper questionnaires kept coming in until August 2021 and these were included in the analyses. In total, 1151 respondents answered the SILQ (691 online and 460 on paper), resulting in a 29% response rate. All paper questionnaires were visually inspected by EK, resulting in the exclusion of five respondents due to a high number of non-responses. The paper questionnaires were then optically scanned. Multiple responses to items in the paper questionnaires were randomized if ticks were made in two adjacent checkboxes (15 respondents).

Based on the Rasch analysis, combined with a theoretical discussion, problematic items in the preliminary version of the SILQ were deleted one by one in an iterative evaluation approach for each of the four scales. Deleted items had demonstrated problems related to local independence or poor item fit in combination with a clear item overlap and/or unclear relation to the underlying construct.

In the survey, we also included background variables and a measure of perceived justice related to interactions with insurance systems [21], which was translated into Swedish to serve as an outcome measure. The results of that analysis are reported in a separate paper [22].

### Data Analysis

The data analysis was conducted in two steps with two different purposes. In the first step, the aim was to use the Rasch model together with theoretical discussions among the author group to select the most important items for the four domains Understand, Obtain, Act, and System from the preliminary version of the SILQ. In the second step, the aim was to evaluate the psychometric properties of each of the four scales that form the final version of the SILQ (see further details in Table 1). For both steps, the unidimensional Rasch measurement model for ordered categories (unrestricted polytomous Rasch model) was used [10]. The Rasch analyses were conducted using RUMM2030 version 5.4 (Rumm Laboratory Pty Ltd, Duncraig, Australia) which use a conditional maximum likelihood estimation procedure. Further, in the estimation of the parameters, the mean location of items is constrained to 0.0 by default. In the present study, the focus was on global and individual item fit, response category functioning, local independence, unidimensionality, person-item threshold distribution, and reliability. In addition, differential item function (DIF) was evaluated for age, sex, education, and data collection method. Detailed information about these aspects of measurement properties, statistical evaluation and interpretation are presented in Table 1 [10, 23–27]. All Rasch analyses were based on a class interval of 10. The significant level was overall set at *p* < 0.05, but Bonferroni corrections were applied in the evaluation of individual item fit in the Rasch analyses (*p* < 0.008 for Obtain, *p* < 0.007 for Understand, and *p* < 0.002 for Act, and System). Bonferroni corrected *p*-values were also applied for evaluations of DIF (*p* < 0.002 for Understand and *p* < 0.003 for Obtained, Act, and System).

**Table 1:** An overview of the Rasch model related to the evaluation of the social insurance literacy questionnaire

Measurement properties	Statistical evaluation	Interpretation
Global fit	Total item trait interaction, chi-square statistics	Evaluates the overall fit between the model and data. The mean of the item fit residual should be close to 0 and the standard deviation of the item fit residual close to 1 for both item and persons. Moreover, the total item trait interaction, chi-square based statistics, should be non-significant [26].
Item fit	Standardized fit residual values and item characteristic curves	Evaluates the fit between the model and data for each item. Standardized fit residual values should be within the range ± 2.5 and Bonferroni corrected *p*-values should be non-significant [25]. The Bonferroni corrected *p*-value depends on the number of items and was therefore set at *p* < 0.008 for the Obtain scale, *p* < 0.007 for the Understand scale, *p* < 0.010 for the Act scale and System scale. The class intervals (i.e., persons with similar ability levels) are expected to follow the assumed line in the item characteristic curves to support model fit. A steeper curve indicates higher discrimination while a flatter curve indicates lower discrimination [13].
Response category function	Ordering of the centralized item thresholds	Evaluates that the response categories correspond to the level of the latent variable, i.e., social insurance literacy. Disordered centralized item thresholds may indicate that the scoring function is not working as intended [25].
Local independency	Item residual correlations	Evaluates that item residuals, i.e., item variance not explained by the latent variable, are not correlated, an indication of multidimensionality. Item residual correlations should be below 0.2 of the mean of all items’ residual correlations [23].
Unidimensionality	Principal component analysis of residuals and the t-test approach	Evaluates that items cover one underlying construct. Items with strongest positive and negative loadings on the first principal component is used to estimate separate person locations (i.e., person measures) and associated standard errors. A series of paired t-test is then conducted to compare person locations based on the two different subsets of items. Fewer than 5% of the t-tests are supposed to be significant (*p* < 0.05), alternatively the lower bound of the Agresi-Coull binominal 95% confidence interval should overlap by 5% to support unidimensionality [24, 27].
Person-item threshold distribution (targeting)	Distribution of item thresholds vs. person ability level.	Evaluates to what extent the item difficulty represents person ability. The mean person location is expected to be around the mean item threshold location, i.e., 0 logits. In addition, the item thresholds are expected to cover about the same range of the logit scale as person locations [25].
Reliability	Person separation index	Evaluates the reliability of the scale. Person separation index are expected to exceed 0.7 to support reliability [25].
Differential item functioning (DIF)	Two-way ANOVA with Bonferroni corrected *p*-values	Evaluates if item responses are biased by external factors, such as groups of different age and sex. Significant main effect of group and interaction effect between group and person location indicate uniform and non-uniform DIF, respectively. In the present study age (< 50 years vs. ≥50 years), sex (male vs. female), education level (university vs. no university), and data collection method (web questionnaires vs. paper questionnaires) were evaluated. Bonferroni corrected p-value depends on the number of items and was therefore set at *p* < 0.002 for the Understand scale and *p* < 0.003 for the Obtained, Act, and System scales. If DIF is presented, an item-split analysis can be conducted to evaluate if the DIF is real of artificial. In this analysis, person locations before and after DIF is solved is compared using paired t-test; a non-significant test indicate artificial DIF while a significant test indicate real DIF [13].

Data quality of the final version of the SILQ was evaluated in terms of item and scale score distributions and missing data patterns, presented as median, quartiles, and percentages. The distribution of the raw scale scores, calculated by adding the item responses in each scale, were also examined using the Shapiro–Wilk test of normality, histogram, and normal QQ plot. These analyses were mainly conducted to detect potential problems with floor and ceiling effects of the raw scores. In addition, an ordinal version of Cronbach’s alpha and traditional Cronbach’s alpha was used to evaluate internal consistency reliability [28]. These analyses were conducted using R, version 4.2.0 (The R Foundation for Statistical Computing, Vienna, Austria), including the following packages: psych 2.2.5, summarytools 1.0.1, and sjmisc 2.8.9.

#### Ethical Considerations

Respondents were informed that answering the survey was voluntary, and that they agreed to participate by answering and submitting the questionnaire. The study was approved by the Swedish Ethical Review Authority (No. 2019-01671). The sharing of clients’ addresses was approved by the SIA’s legal department after reviewing the project’s ethical approval.

## Results

### Characteristics of Participants

The final sample included 1151 persons, 72% females and 28% males. The mean age was 48.4 (SD = 10.9) years. Most of the participants were born in Sweden (*n* = 989, 87%) and about half of them had a university degree (*n* = 545, 48%). About half of the participants had been on sick leave for more than one year (*n* = 593, 52%) (Table 2). Of the respondents, 755 had an ongoing sick leave case, and 396 had withdrawn benefits. A descriptive non-response analysis was carried out based on information from the SIA about the demographics of responders and non-responders. This analysis showed similar patterns in the demographics for both groups, although the respondents to a larger degree were female (72% vs. 65%), slightly older (49 vs. 46 years), having higher educational levels (50% vs. 32% with post-secondary education), and being native Swedes (83% vs. 75%), relative to non-respondents.

**Table 2:** Study participants (*n* = 1151)

Age (years), mean (SD) [range]	48.4 (10.9) [20–65]
Sex, *n* (%)	
Female	827 (72.0%)
Male	322 (28.0%)
Missing	2
Swedish born, *n* (%)	
Yes	989 (86.7)
No	152 (13.3)
Missing	8
University education, *n* (%)	
Yes	545 (47.7)
No	598 (52.3)
Missing	8
Duration of current sick leave spell at the time of recruitment, *n* (%)	
No current sick leave	214 (18.6%)
1–90 days	8 (0.7%)
91–180 days	179 (15.6%)
181–365 days	157 (13.6%)
1–2 years	359 (31.2%)
2–5 years	232 (20.2%)
> 5 years	2 (0.2%)
SILQ raw scores, Mdn (IQR) [min–max]	
Obtain scale (possible range 0–18)	8 (5, 11) [0–18]
Understand scale (possible range 0–21)	8 (5, 11) [0–21]
Act scale (possible range 0–15)	7 (4, 9) [0–15]
System scale (possible range 0–15)	9 (5, 12) [0–15]

*IQR* Interquartile range, *Mdn* Median, *SD* Standard deviation, *SILQ* Social Insurance Literacy Questionnaire

### Psychometric Evaluation of the Final Version of the SILQ

The final version of the SILQ (see Supplement 1) consisted of 23 items, of which 6 in the Obtain scale, 7 in the Understand scale, 5 in the Act scale, and 5 in the System scale. The scale scores are calculated by summing the item responses in each scale. Therefore, the possible score range is 0–18 for the Obtain scale, 0–21 for the Understand scale, and 0–15 for the Act and System scales. It is also possible to transform the raw scores into logit scores using the conversion table in Supplement 2.

### Item and Scale Score Statistics

The median score ranged between 0 and 2 for the items in the four scales. All response options were used and no pronounced problems with floor and ceiling effects were shown. The amount of missing data was low and ranged between 0.2% and 1.3%. The response option ‘Don’t know/not relevant’ ranged between 1.4% and 27.8%, and was highest for item 9 (26.6%, ‘With help from others understand information from insurance agency’) in the Understand scale, item 2 (23.7%, ‘With help from others get information’) in the Obtain scale, and items 17 (22.5%, ‘Get help from others to argue for your case’) and 18 (27.8%, ‘Appeal decisions’) in the Act scale (Table 3). For these items, a larger number of N/A answers are expected as they are not relevant for people living in single-person households, or who have not appealed their decisions.

**Table 3:** Item statistics of the social insurance literacy questionnaire for the four scales (*n* = 1151)

Scales and item content	Mdn (IQR)	Score distribution, %					
		Very good (0)	Good (1)	Bad (2)	Very bad (3)	Don’t know/not relevant	Missing
Obtaining information (How do you rate your ability to get…)							
1 Information from insurance agency	1 (1, 2)	20.7	48.3	20.9	7.1	2.8	0.3
2 Information with help from others	1 (0, 1)	21.7	36.5	12.3	4.9	23.7	0.9
3 Information about possibilities to influence case	1 (1, 2)	17.6	28.0	24.0	18.1	11.5	0.9
4 Information about other actors’ roles	1 (1, 2)	20.9	38.1	23.9	9.6	6.4	1.1
5 Information about laws and regulations	2 (1, 2)	15.6	29.2	27.9	19.2	7.0	1.1
6 Clarifications about decisions	1 (1, 2)	17.4	27.8	22.3	12.5	18.7	1.3
Understanding information (How do you rate your ability to understand…)							
7 How to fill in forms	1 (1, 2)	18.9	43.4	22.1	10.2	5.1	0.4
8 Spoken information from insurance agency	1 (0, 1)	30.5	41.2	16.0	7.0	5.0	0.4
9 Information from insurance agency with help from others	1 (0, 1)	19.8	35.5	12.6	4.9	26.6	0.7
10 What information to supply to insurance agency	1 (0, 2)	26.0	43.6	17.3	9.4	3.4	0.4
11 At what times to supply information to insurance agency	1 (0, 1)	38.4	38.6	12.9	5.6	4.0	0.6
12 Laws and regulations related to case	2 (1, 2)	13.8	29.4	29.5	19.5	6.7	1.1
13 Decisions from insurance agency	1 (0, 1)	30.5	44.2	13.6	9.6	1.4	0.7
Act on information (How do you rate your ability to…)							
14 Ask questions if more information is needed	1 (0, 2)	26.2	39.6	17.9	9.2	6.6	0.5
15 Deliver information on time	0 (0, 1)	49.7	35.9	7.6	2.7	3.7	0.5
16 Argue by referring to laws, regulations or certificates	2 (1, 3)	12.2	22.0	26.6	22.2	16.2	1.0
17 Get help from others to argue for your case	1 (1, 2)	16.9	29.3	18.9	11.6	22.5	1.0
18 Appeal decisions	2 (1, 3)	12.3	17.3	18.6	22.9	27.8	1.2
System comprehensibility (How do you rate the insurance agency’s ability to/think that the staff succeeds in…)							
19 Offer information you understand	1 (1, 2)	13.0	41.9	24.8	16.9	3.0	0.4
20 Make decisions within a reasonable time	1 (1, 2)	14.3	38.3	20.5	22.2	3.9	0.7
21 Clearly explain reasons for decisions	2 (1, 3)	11.8	31.5	22.6	26.9	6.6	0.6
22 Being available	1 (1, 2)	11.4	36.3	25.5	18.7	8.0	0.2
23 Showing that they trust you	2 (1, 3)	17.8	23.1	16.3	34.2	8.0	0.5

*Mdn* Median, *IQR* Interquartile range

After the response option ‘Don’t know/not relevant’ was recoded as missing data, the share of computable subscale scores, without imputation, was highest for the System scale (*n* = 904, 78.5%), Understand scale (*n* = 732, 63.6%), Obtain scale (*n* = 685, 59.5%), and Act scale (*n* = 672, 58.4). Based on the Shapiro–Wilk test of normality, the score distribution for the subscales deviated significantly from a normal distribution (*p* < 0.001). Graphically, the histograms and normal QQ plots showed that the subscale scores for the Obtain, Understand, and Act scales were well spread and followed a normal distribution, except at the lower end of the scales. Therefore, no problems with floor and ceiling effects were shown. The System scale demonstrated a more uniform distribution. All scales peaked at between 2 and 3 reflected in the quartiles which are presented in Table 3.

### Global Fit

The total item trait interaction chi-square statistics showed that all scales deviated significantly from the Rasch model (*ps* = 0.012 to < 0.001). The mean of the item fit residual value was close to 0 for the Act scale (0.002). The rest of the scales deviated only slightly from this expected value: 0.093 for the Understand scale, − 0.119 for the Obtain scale, and − 0.255 for the System scale. The Understand scale had a standard deviation of the item fit residual close to 1, the standard deviation for the other scales were above 1 (Table 4).

**Table 4:** Global fit statistics and reliability for the social insurance literacy questionnaire (*n* = 1151)

	Scales			
	Obtain	Understand	Act	System
Items				
Location, mean	0.000	0.000	0.000	0.000
Location, SD	0.518	0.557	0.905	0.173
Fit residual, mean	− 0.119	0.093	0.002	− 0.255
Fit residual, SD	1.643	0.956	2.292	2.296
Persons				
Location, mean	− 0.483	− 0.817	− 0.396	0.204
Location, SD	1.762	1.703	1.521	1.877
Fit residual, mean	− 0.569	− 0.473	− 0.398	− 0.555
Fit residual, SD	1.322	1.302	0.953	1.461
Total item trait interaction				
Total item *χ*^2^	80.35	112.56	110.18	92.39
df	54	63	45	45
* p*-value	0.012	< 0.001	< 0.001	< 0.001
Reliability				
Person separation index	0.80	0.82	0.68	0.81
Ordinal alpha	0.91	0.91	0.86	0.91
Cronbach’s alpha	0.82	0.83	0.75	0.84

SD = Standard deviation, df = Degrees of freedom

Since the chi-square test of fit is highly dependent on the sample size, RUMM2030 allows to evaluate the chi-square statistics using a changed effective sample size without changing the data or any other equations, except in the formula of the chi-square [29]. Therefore, to evaluate the effect of the sample size on global fit, the item trait interaction *χ*^2^ statistics were also evaluated by changing the effective sample to 500. In these sensitivity analyses, none of the SILQ scales deviated significantly from the model: Obtain *χ*^2^(54) = 38.6, *p* = 0.943; Understand *χ*^2^(63) = 53.3, *p* = 0.802; Act *χ*^2^(45) = 54.5, *p* = 0.156; System *χ*^2^(45) = 44.8, *p* = 0.482.

### Individual Item Fit

The standardized individual item fit residual and chi-square goodness-of-fit statistics are presented in Table 5. According to the residual fit statistics, all items demonstrated good fit to the Rasch model except three; item 16 in the Act scale, and items 21 and 22 in the System scale. In addition to this, items 10 and 12 in the Understand scale and item 23 in the System scale deviated significantly from the model, but with acceptable residual fit statistics. A graphical inspection of the ICC for these items (Supplement 3) showed a good agreement between data and the Rash model for items 10, 12, and 22. Regarding items 16, 21, and 23, persons had a steeper curve than expected according to the model, but the deviations were minor.

**Table 5:** Item location, item fit statistics, and item thresholds for the items in the social insurance literacy questionnaire (*n* = 1151)

Items^a^	Item location	Residual^b^	Item fit statistics		Centralized item thresholds			
			χ^2c^	*p*-value^d^	I	II	III	Disordered thresholds
Obtain scale								
5 Information about laws and regulations	− 0.61	− 1.62	18.11	0.034	− 1.83	0.20	1.63	No
3 Information about possibilities to influence case	− 0.44	− 2.29	18.18	0.033	− 1.71	0.27	1.44	No
6 Clarifications about decisions	− 0.17	0.40	9.13	0.425	− 1.76	0.10	1.66	No
4 Information about other actors’ roles	0.11	0.11	7.88	0.547	− 2.11	0.21	1.90	No
1 Information from insurance agency	0.32	0.36	11.59	0.237	− 2.53	0.49	2.04	No
2 Information with help from others	0.79	2.32	15.47	0.079	− 2.18	0.55	1.63	No
Understand scale								
12 Laws and regulations related to case	− 1.04	1.53	28.13	**< 0.001**	− 1.89	0.22	1.67	No
7 How to fill in forms	− 0.36	− 0.21	11.08	0.270	− 2.40	0.64	1.76	No
10 What information to supply to insurance agency	− 0.03	− 0.95	25.20	**0.003**	− 2.06	0.68	1.38	No
13 Decisions from insurance agency	0.11	0.68	4.56	0.871	− 1.84	0.86	0.98	No
8 Spoken information from insurance agency	0.27	− 0.79	21.24	0.012	− 1.92	0.47	1.46	No
9 Information from insurance agency with help from others	0.42	0.94	12.75	0.174	− 2.15	0.59	1.55	No
11 At what times to supply information to insurance agency	0.63	− 0.55	9.61	0.383	− 1.67	0.45	1.22	No
Act scale								
16 Argue by referring to laws, regulations or certificates	− 0.85	**− 3.30**	46.71	**< 0.001**	− 1.69	0.21	1.48	No
18 Appeal decisions	− 0.79	− 1.17	19.65	0.020	− 1.25	0.26	0.99	No
17 Get help from others to argue for your case	− 0.03	0.36	13.77	0.131	− 1.67	0.38	1.29	No
14 Ask questions if more information is needed	0.31	1.76	16.29	0.061	− 1.71	0.50	1.21	No
15 Deliver information on time	1.36	2.36	13.77	0.131	− 1.45	0.61	0.84	No
System scale								
21 Clearly explain reasons for decisions	− 0.22	**− 3.04**	32.82	**< 0.001**	− 2.22	0.72	1.50	No
23 Showing that they trust you	− 0.12	− 2.15	21.86	**0.009**	− 1.29	0.63	0.66	No
22 Being available	0.05	**2.56**	5.47	0.792	− 2.54	0.74	1.80	No
20 Make decisions within a reasonable time	0.07	0.55	17.98	0.035	− 2.26	0.89	1.36	No
19 Offer information you understand	0.22	0.81	14.26	0.113	− 2.59	0.71	1.89	No

^a^Items are sorted in location order, from the easiest to the most difficult

^b^Standardized individual item fit residuals ± 2.5 are marked with bold

^c^ χ^2^ values, all with 9 degrees of freedom

^d^The Bonferroni corrected *p*-values are *p* < 0.008 for the Obtain scale, *p* < 0.007 for the Understand scale, *p* < 0.010 for the Act scale, and *p* < 0.010 for the System scale. Significant values are marked with bold

### Response Category Functioning

No disordered centralized item thresholds were detected for any of the items in the SILQ (Table 5).

### Person-Item Threshold Distribution

**Fig. 1:**
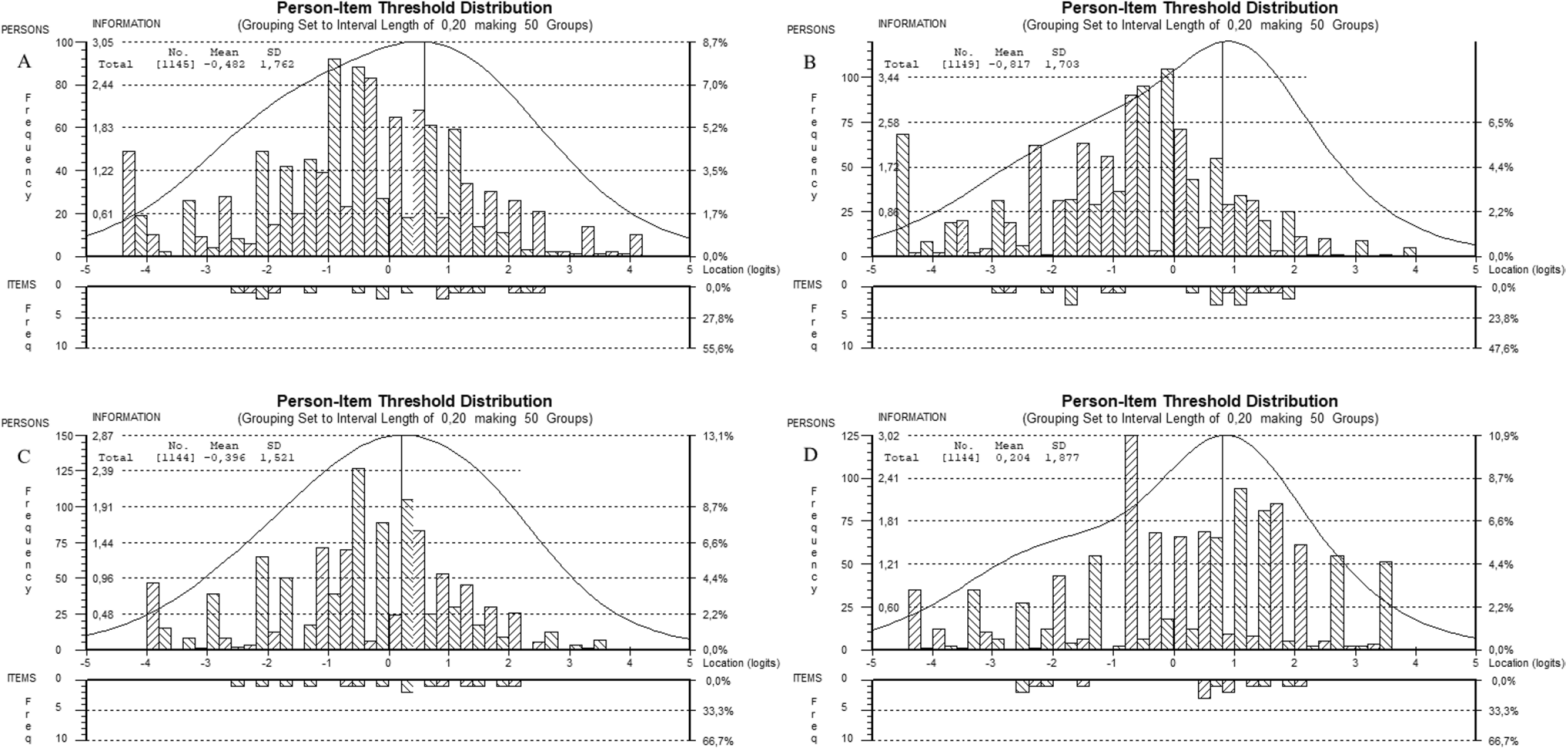
The person-item threshold distribution for the SILQ scales: **A** Obtain, **B** understand, **C** act, and **D** system

The person-item threshold distribution for SILQ subscales is shown in Fig. 1A through D. All subscales covered at least around − 2.5 to 2.0 logits of the person ability scores; the Obtain scale covered up to 2.5 while the Understand scale covered down to − 3.0. In addition, the Understand scale and the System scale had a gap between around 0 and − 1. Thus, persons with very low or very high levels of social insurance literacy were not well targeted by the items in any of the SILQ scales.

### Unidimensionality

The t-test approach, comparing person location based on two subset of items from the principal component analysis of residuals, supported unidimensionality since fewer than 5% of the participants had significantly different scores (*p* < 0.05) on the two subset of items; 46 (4.0%) for the Understand scale, 35 (3.2%) for the Obtain scale, 42 (4.1%) for the Act scale, and 41 (3.6%) for the System scale.

### Local Independency

The mean item residual correlations were − 0.25 for the Obtain scale, − 0.17 for the Understand scale, − 0.27 for the Act scale, and − 0.25 for the System scale. Thus, the critical values were − 0.02, 0.03, − 0.07, and − 0.05, respectively, for the scales. Based on these critical values, problems with local dependency were only detected between items in the Act scale; item 16 and 17 (− 0.04) and item 16 and item 18 (− 0.05).

### Reliability

The person separation index was highest for the Understand scale (0.82), followed by the System scale (0.81), Obtain scale (0.80), and Act scale (0.68). The ordinal alpha ranged between 0.86 and 0.91, and Cronbach’s alpha ranged between 0.75 and 0.84 (Table 4).

### Differential Item Functioning

Item 10 in the Understand scale and item 20 in the System scale demonstrated uniform DIF for education level while item 16 in the Act scale demonstrated uniform DIF for sex. In item 10, persons without university degree tended to score significantly lower levels than persons with university degree. The opposite finding was shown for item 20. In item 16, males tended to score significantly lower levels compared to females (Table 6, Supplement 4). When comparing the person location scores (logits) before and after the DIF was solved, significant differences were shown. The mean differences before and after item 10 was split for education was 0.004 [*t*(1148) = 4.06, *p* < 0.001, *d* = 0.12]. The corresponding mean difference for item 20 was 0.006 [*t*(1148) = 4.06, *p* < 0.001, *d* = 0.09]. Thus, both item 10 and item 20 had a minor effect size on the person location scores. In contrast, the mean differences before and after item 16 was split for sex was 0.105 [*t*(1148) = 59.27, *p* < 0.567, *d* = 1.75], which represent a large effect size. No problems with DIF were detected for age or data collection method.

**Table 6:** Probability values for the evaluation of uniform and non-uniform differential item functioning (DIF) for age, sex, education, and data collection method

Scales	Items	Age		Sex		Education		Data collection method	
		Uniform	Non- uniform	Uniform	Non- uniform	Uniform	Non- uniform	Uniform	Non- uniform
Obtain	1	0.523	0.830	0.326	0.502	0.418	0.172	0.320	0.120
	2	0.119	0.180	0.035	0.911	0.658	0.472	0.720	0.955
	3	0.240	0.290	0.056	0.271	0.238	0.465	0.181	0.471
	4	0.004	0.208	0.089	0.014	0.755	0.023	0.351	0.774
	5	0.268	0.684	0.250	0.704	0.017	0.777	0.163	0.312
	6	0.373	0.125	0.053	0.942	0.980	0.939	0.565	0.902
Understand	7	0.085	0.684	0.104	0.998	0.584	0.238	0.852	0.629
	8	0.071	0.160	0.195	0.126	0.223	0.932	0.715	0.326
	9	0.169	0.523	0.510	0.611	0.844	0.015	0.418	0.524
	10	0.262	0.694	0.742	0.470	**0.001**	0.233	0.344	0.618
	11	0.101	0.810	0.052	0.734	0.135	0.491	0.021	0.860
	12	0.508	0.475	0.278	0.503	0.149	0.276	0.959	0.373
	13	0.015	0.515	0.444	0.985	0.048	0.979	0.870	0.699
Act	14	0.499	0.688	0.035	0.484	0.031	0.363	0.591	0.728
	15	0.200	0.215	0.040	0.466	0.965	0.287	0.104	0.293
	16	0.008	0.937	**< 0.001**	0.717	0.525	0.685	0.043	0.889
	17	0.834	0.698	0.412	0.229	0.465	0.186	0.070	0.298
	18	0.930	0.333	1.000	0.883	0.657	0.193	0.615	0.096
System	19	0.020	0.173	0.190	0.162	0.056	0.096	0.094	0.584
	20	0.029	0.111	0.017	0.611	**< 0.001**	0.244	0.578	0.117
	21	0.938	0.176	0.122	0.730	0.015	0.143	0.862	0.597
	22	0.898	0.245	0.930	0.869	0.582	0.334	0.666	0.066
	23	0.646	0.132	0.682	0.950	0.347	0.415	0.298	0.681

The DIF analysis is based on a two-way ANOVA with Bonferroni corrected *p*-values; *p* < 0.002 for the understand scale and *p* < 0.003 for the obtained, act, and system scales

## Discussion

This study describes the development of the SILQ, and the psychometric properties based on Rasch measurement theory. The results support that SILQ is a multidimensional measure, covering four important theoretical aspects of literacy in the social insurance field: obtaining, understanding, and acting on information, and system comprehensibility. Overall, the SILQ scales demonstrated satisfactory fit according to the Rasch model.

### Psychometric Properties of the SILQ

The number of item nonresponse was low (except for items that were not applicable for everyone), which may indicate that the items were considered important and easy to answer. This conclusion is supported by the cognitive interviews, where the items were generally considered understandable. Some items may not be relevant to all respondents such as using help from others or appeal decisions. These aspects were, however, considered important to include from a theoretical perspective and instead of using more complicated item types such as filter questions respondents are offered a response option ‘not relevant.’ As the response option ‘Don’t know/not relevant’ needed to be converted to missing data, the share of computable subscales scores without imputations were heavily affected. To handle this, we recommend users of the SILQ to calculate the mean scores by dividing with the number of completed items, as long as more than half of the items in the scale is completed.

Although the total item trait interaction *χ*^2^ based statistics indicated a misfit for all SILQ scales, the item fit was overall good. In fact, only one item showed standardized fit residuals outside the expected range of ± 2.5 but without a significant deviation from the model (item 22), three items deviated significantly from the model but had acceptable standardized fit residuals (items 10, 12, and 23), and two items had both a large standardized fit residual and deviated significantly from the model (items 16 and 21). The graphical examination of the item characteristic curves for these items showed that items 10, 12, and 22 conformed well with the Rasch model while items 16, 21, and 23 had a somewhat steeper ICC than expected, i.e., to high discrimination ability [30]. It should be recognized that the graphical deviations were minor and that the *χ*^2^ based statistics are highly affected by large sample sizes. Thus, the large sample size may explain why the total item trait interaction was statistically significant and why three items deviated significantly from the model despite acceptable standardized fit residuals. This explanation is also supported by sensitivity analyses of the total item trait interaction chi-square statistics, using the changed effective sample procedure; none of the SILQ scales deviated significantly from the Rasch model in these sensitivity analyses. Based on these findings, it seems that the Obtain scale and Understand scale conform well with the Rasch model while the Act scale and System scale included one and two items that not conformed perfectly with the Rasch model.

All scales in the SILQ seems to be unidimensional according to the principal component analysis of residuals and the t-test approach. Unidimensionality is an important assumption in the Rasch model, but also in classical test theory, and implies that the items cover only one underlying latent variable. An item is considered unidimensional if the systematic differences within the item variance are only due to the latent variable. Thus, a set of items is seen as unidimensional if there are no correlated residuals between the items once the variance due to the latent construct is controlled [31]. Therefore, local independence is also an important aspect to examine in the evaluation of unidimensionality. The examination of local independence showed that the item residual correlations were higher than expected between items 16 and 17, and 16 and 18 in the Act scale. Despite these correlations being higher than expected, they were still very low and negative. Therefore, they did not indicate any severe problems with local independence. However, item 16 should be explored further in future validation studies.

Another important finding is that the response scale seems to work as expected since no problems with reversed thresholds were detected. In addition, all scales demonstrated satisfactory reliability measured with person separation index and ordinal alpha. One exception is the Act scale that had a person separation index below but close to the expected value of > 0.7. Both the coefficient alpha and person separation index can be interpreted in the same way, the only difference is that coefficient alpha is based on the raw scores while person separation index is based on the logit scores [32]. Thus, this may explain the large difference between the alpha coefficients and the person separation in this scale.

The evaluation of person-item threshold distribution (i.e., targeting) showed that the SILQ scales are sensitive and capture different levels of social insurance literacy, except for persons with very high and very low levels, that are not well targeted by the items. In future revisions, inclusion of more easy and more difficult items should be considered. From a practical perspective, more difficult items that reflect those with lowest levels of social insurance literacy (i.e., high scores on SILQ) seems to be most important since people with high social insurance literacy is seldom a risk group or target for interventions.

The number of items with DIF was small but the comparison of person location scores before and after DIF was solved showed a significant difference, which supported real DIF in these three items. However, it should be recognized that the effect size was small for the DIF related to education level in item 10 and 20. However, the DIF for sex in item 16 had a large effect on the person location scores. Therefore, users of the SILQ can overall make invariant comparisons between groups of different age, sex, and education levels. One exception is the Act scale where differences between women and men should be interpreted with carefulness.

### Theoretical Implications

Social insurance literacy is a complex phenomenon since it contains multiple dimensions and is likely to vary between contexts and points in time. Overall, however, the study results indicate that the SILQ functions as a measure for the four domains of the social insurance literacy concept. The SILQ is, compared to established concepts such as health literacy, designed to study a more specific type of literacy relevant in interactions within social insurance contexts, which makes its uses narrower, but simultaneously more adequate for use in these contexts. Although partly overlapping with concepts such as administrative burden or literacy, it is more catered to the types of relationships between actors in this setting, where clients are likely to have decreased abilities. It is also more focused on the individual-contextual relationship by including a domain focused exclusively on the insurance system.

It is central to point out that social insurance literacy comprises both individual and contextual aspects, and that the literacy measured with the SILQ does not capture a static property among individuals. The system comprehensibility sub-scale is therefore as central to the overall assessment as the individual abilities, and they are also likely to interact—where the system representatives communicate better, the individual abilities may have lesser significance (or may even improve). The SILQ should hence be used as a measure that includes all four domains, to minimize the risk of over-interpreting it as a measure of individual capacities. Interpretations need to account for these potential interaction effects, bearing in mind that the SILQ gives a rather crude indication of such aspects. SILQ scorings are further highly influenced by the legal and cultural context which needs to be accounted for in interpretations of results.

A potential development of the current study could be focused on inter-relatedness between the domains, comparative studies between populations and contexts, and mixed methods approaches where the SILQ is combined with qualitative methods, which would be useful to complement the interpretation of the measurements. Future research could also focus on how system representatives experience their individual communication abilities, and how social insurance literacy is influenced by medical conditions or functional disabilities, and by interactions between system representatives and clients. It would also be interesting to analyze overlap, similarities, and differences between the SILQ and other measures, such as health literacy or administrative literacy, in empirical studies.

#### Methodological Considerations

Self-assessments were used both of respondents’ abilities and of the system’s comprehensibility, which may have both advantages and limitations. Subjective assessments of system comprehensibility capture how the communication is experienced, which is likely a relevant measure of whether the information comes across and is understandable. For the individual abilities, subjective assessments may be less reliable than actual tests of those abilities, which means that it is not possible to determine whether the stated ability reflects an actual ability. Overall, however, the results suggest that social insurance literacy is measurable on a subjective level.

The sample of people on sick leave was considered relevant due to their experience of communication with social insurance authorities. The response rate was 29%, which is a relatively expected rate considering this sample as people on sick leave may have limited energy and ability to respond to surveys. To manage this, the sample was made large enough to secure a sufficient number of responses for the validation analyses. No generalization claims are made in this analysis—the purpose was solely to test how well the questionnaire works in the population.

The SILQ was developed as a multidimensional scale, covering four different domains of social insurance literacy. However, the Rasch model requires unidimensionality [10]. Therefore, the SILQ scales were treated as separate in the analyses. Further research could perform additional analyses to explore whether the three individual domains could constitute a unidimensional construct.

One important limitation is that the design of the present study did not allow for any evaluations of criterion validity, convergent and divergent validity, or test–retest reliability and responsiveness to change. These aspects of validity are not addressed by the Rasch model and needs therefore to be considered in future evaluations of the SILQ. Another limitation is that we did not perform any cross-cultural adaptations [33]. The questionnaire was back translated for linguistical accuracy and is currently worded to allow for use in different insurance systems, but since the systems vary considerably across jurisdictions, it would be useful to test different versions in different contexts. The survey was sent out during the Covid-19 pandemic. While this may have influenced sick leave, our sample would remain mainly unaffected since they at the time of sampling had been on sick leave for at least 180 days, meaning before the onset of the pandemic. What could possibly have had an impact on the study is an increased burden on the SIA during this period. We could see no clear effects of the pandemic in the results, including the responses to open questions.

Finally, the SILQ scales seems to conform well with the Rasch model. However, item 16 should be considered in future revisions of the instrument since it demonstrated some problems with item fit, showing response dependence with two other items in the Act scale, and demonstrated DIF for sex.

## Conclusion

This study is a first step toward an exploration of literacy in the social insurance field. The SILQ covers both individual capacities and aspects related to systems’ ability to communicate in a comprehensible way, and the results from this study show that the SILQ has acceptable psychometric properties according to the Rasch model. It may therefore be used to further develop research on literacy, legitimacy, and comprehensibility in social insurance systems, as well as by agencies to evaluate the adequacy of their communication.

### Supplementary Information

Below is the link to the electronic supplementary material.

Supplementary material 1 (DOCX 32 kb)

Supplementary material 2 (DOCX 20 kb)

Supplementary material 3 (DOCX 153 kb)

Supplementary material 4 (DOCX 121 kb)
