# Supplementary material for: The Social Insurance Literacy Questionnaire (SILQ): Development and Psychometric Evaluation
Source: J Occup Rehabil. 2023 Dec 30;34(3):693–706. doi: 10.1007/s10926-023-10159-7 (PMC11364705; doi:10.1007/s10926-023-10159-7)
Supplement: Supplementary file 1 — Supplementary material 1 (DOCX 32 kb) [file 10926_2023_10159_MOESM1_ESM.docx]

**Supplement 1: The Social Insurance Literacy Questionnaire (SILQ)**

*Instruction to users of the SILQ*

This questionnaire aims to measure social insurance literacy, which is defined as follows:

Social insurance literacy is the extent to which individuals can obtain, understand and act on information in a social insurance system, related to the comprehensibility of the information provided by the system.

The SILQ contains questions about 1) individuals’ views on their own abilities to obtain, understand and act on information, and 2) individuals’ perspective on the comprehensibility of the social insurance system.

The layout can be adapted to suit the target survey, where questions may be divided into matrices, and the numbering of questions can be adapted to fit the design. The order of questions should however be kept intact.

In this document, a placeholder (XX) is used to indicate the social insurance organization in question, which should be replaced with its proper name. “Social insurance”, “sick leave” and other similar terminology may also be changed to fit the context (e.g., to “workers’ compensation”).

## Your ability to act in the social insurance system

These questions are about your own ability to make contact with XX, and to understand the social insurance system. The questions relate to your current or latest sick leave period. Answer the questions based on how you experience these things today.

## Obtaining information

**How do you rate your ability to…**

|  |  | Very good | Rather good | Rather bad | Very bad |  | Don’t know/not relevant |
| --- | --- | --- | --- | --- | --- | --- | --- |
| 1. | …get the information you need from XX? | 🞎 | 🞎 | 🞎 | 🞎 |  | 🞎 |
| 2. | …with help from others get the information you need (e.g., relatives, others on sick leave, health care professionals, employers, union representatives)? | 🞎 | 🞎 | 🞎 | 🞎 |  | 🞎 |
| 3. | …get information about your possibilities to influence your sick leave case? | 🞎 | 🞎 | 🞎 | 🞎 |  | 🞎 |
| 4. | …get information about other actors’ roles in your sick leave case (e.g., healthcare, employers, union representatives, authorities)? | 🞎 | 🞎 | 🞎 | 🞎 |  | 🞎 |
| 5. | …get information about laws and regulations? | 🞎 | 🞎 | 🞎 | 🞎 |  | 🞎 |
| 6. | …get clarifications about decisions in your sick leave case if necessary? | 🞎 | 🞎 | 🞎 | 🞎 |  | 🞎 |

## Understanding information

**How do you rate your ability to…**

| 7. | …understand how to fill in forms? | 🞎 | 🞎 | 🞎 | 🞎 |  | 🞎 |
| --- | --- | --- | --- | --- | --- | --- | --- |
| 8. | …understand spoken information from staff at XX? | 🞎 | 🞎 | 🞎 | 🞎 |  | 🞎 |
| 9. | …with help from others understand information from XX (e.g., relatives, others on sick leave, health care professionals, employers, union representatives)? | 🞎 | 🞎 | 🞎 | 🞎 |  | 🞎 |
| 10. | …understand what information you are expected to supply to XX? | 🞎 | 🞎 | 🞎 | 🞎 |  | 🞎 |
| 11. | …understand at what times you need to supply information to XX? | 🞎 | 🞎 | 🞎 | 🞎 |  | 🞎 |
| 12. | …understand the laws and regulations related to your sick leave case? | 🞎 | 🞎 | 🞎 | 🞎 |  | 🞎 |
| 13. | …understand decisions from XX? | 🞎 | 🞎 | 🞎 | 🞎 |  | 🞎 |

## Acting on information

**How do you rate your ability to…**

| 14. | …ask questions if you need more information? | 🞎 | 🞎 | 🞎 | 🞎 |  | 🞎 |
| --- | --- | --- | --- | --- | --- | --- | --- |
| 15. | …deliver information (e.g., medical certificates) to XX on time? | 🞎 | 🞎 | 🞎 | 🞎 |  | 🞎 |
| 16. | …argue for your case by referring to laws, regulations or certificates? | 🞎 | 🞎 | 🞎 | 🞎 |  | 🞎 |
| 17. | …get help from others to argue for your case (e.g., relatives, others on sick leave, health care professionals, employers, union representatives)? | 🞎 | 🞎 | 🞎 | 🞎 |  | 🞎 |
| 18. | …formally appeal decisions if you think it is wrong (e.g., to an independent tribunal or a court)? | 🞎 | 🞎 | 🞎 | 🞎 |  | 🞎 |

## Comprehensibility of the social insurance system

These questions concern how easy or hard you think it is to understand the social insurance system. The questions relate to your current or latest sick leave period. Answer the questions based on how you experience these things today.

**As a whole, how do you rate XX’s ability to…**

|  |  | Very good | Rather good | Rather bad | Very bad |  | Don’t know/not relevant |
| --- | --- | --- | --- | --- | --- | --- | --- |
| 19. | …offer information that you can understand? | 🞎 | 🞎 | 🞎 | 🞎 |  | 🞎 |
| 20. | …make decisions within a reasonable time? | 🞎 | 🞎 | 🞎 | 🞎 |  | 🞎 |
| 21. | …clearly explain the reasons for decisions? | 🞎 | 🞎 | 🞎 | 🞎 |  | 🞎 |

**How do you think that the staff at XX succeeds in…**

|  |  | Very good | Rather good | Rather bad | Very bad |  | Don’t know/not relevant |
| --- | --- | --- | --- | --- | --- | --- | --- |
| 22. | …being available when you need to get in touch with them? | 🞎 | 🞎 | 🞎 | 🞎 |  | 🞎 |
| 23. | …showing that they trust you and what you tell them? | 🞎 | 🞎 | 🞎 | 🞎 |  | 🞎 |
