# Supplementary material for: The Social Insurance Literacy Questionnaire (SILQ): Development and Psychometric Evaluation
Source: J Occup Rehabil. 2023 Dec 30;34(3):693–706. doi: 10.1007/s10926-023-10159-7 (PMC11364705; doi:10.1007/s10926-023-10159-7)
Supplement: Supplementary file 2 — Supplementary material 2 (DOCX 20 kb) [file 10926_2023_10159_MOESM2_ESM.docx]

**Supplement 2.** From raw scores to logit scores, conversion table for the SILQ scales.

|  |  |  |  |  |  |  |  |  |  |  |
| --- | --- | --- | --- | --- | --- | --- | --- | --- | --- | --- |
|  |  |  |  |  |  |  |  |  |  |  |
|  |  |  |  |  |  |  |  |  |  |  |
| Obtain | |  | Understand | |  | Act | |  | System | |
|  |  |  |  |  |  |  |  |  |  |  |
|  |  |  |  |  |  |  |  |  |  |  |
| Raw | Logit |  | Raw | Logit |  | Raw | Logit |  | Raw | Logit |
|  |  |  |  |  |  |  |  |  |  |  |
|  |  |  |  |  |  |  |  |  |  |  |
| 0 | -4.251 |  | 0 | -4.527 |  | 0 | -3.844 |  | 0 | -4.226 |
| 1 | -3.374 |  | 1 | -3.619 |  | 1 | -2.903 |  | 1 | -3.333 |
| 2 | -2.704 |  | 2 | -2.927 |  | 2 | -2.189 |  | 2 | -2.577 |
| 3 | -2.192 |  | 3 | -2.4 |  | 3 | -1.649 |  | 3 | -1.936 |
| 4 | -1.748 |  | 4 | -1.948 |  | 4 | -1.197 |  | 4 | -1.316 |
| 5 | -1.34 |  | 5 | -1.539 |  | 5 | -0.8 |  | 5 | -0.733 |
| 6 | -0.956 |  | 6 | -1.157 |  | 6 | -0.436 |  | 6 | -0.253 |
| 7 | -0.592 |  | 7 | -0.799 |  | 7 | -0.091 |  | 7 | 0.134 |
| 8 | -0.246 |  | 8 | -0.466 |  | 8 | 0.245 |  | 8 | 0.469 |
| 9 | 0.084 |  | 9 | -0.161 |  | 9 | 0.582 |  | 9 | 0.779 |
| 10 | 0.405 |  | 10 | 0.118 |  | 10 | 0.927 |  | 10 | 1.085 |
| 11 | 0.722 |  | 11 | 0.375 |  | 11 | 1.289 |  | 11 | 1.403 |
| 12 | 1.043 |  | 12 | 0.617 |  | 12 | 1.681 |  | 12 | 1.753 |
| 13 | 1.374 |  | 13 | 0.848 |  | 13 | 2.126 |  | 13 | 2.174 |
| 14 | 1.727 |  | 14 | 1.077 |  | 14 | 2.69 |  | 14 | 2.755 |
| 15 | 2.118 |  | 15 | 1.309 |  | 15 | 3.405 |  | 15 | 3.556 |
| 16 | 2.582 |  | 16 | 1.553 |  |  |  |  |  |  |
| 17 | 3.208 |  | 17 | 1.819 |  |  |  |  |  |  |
| 18 | 4.054 |  | 18 | 2.127 |  |  |  |  |  |  |
|  |  |  | 19 | 2.509 |  |  |  |  |  |  |
|  |  |  | 20 | 3.055 |  |  |  |  |  |  |
|  |  |  | 21 | 3.834 |  |  |  |  |  |  |
|  |  |  |  |  |  |  |  |  |  |  |
|  |  |  |  |  |  |  |  |  |  |  |
|  |  |  |  |  |  |  |  |  |  |  |
|  |  |  |  |  |  |  |  |  |  |  |
