# Supplementary material for: The Social Insurance Literacy Questionnaire (SILQ): Development and Psychometric Evaluation
Source: J Occup Rehabil. 2023 Dec 30;34(3):693–706. doi: 10.1007/s10926-023-10159-7 (PMC11364705; doi:10.1007/s10926-023-10159-7)
Supplement: Supplementary file 3 — Supplementary material 3 (DOCX 153 kb) [file 10926_2023_10159_MOESM3_ESM.docx]

**Supplement 3:** Item characteristic curves (ICC) for items with standardized fit residual values outside the range of ± 2.5 and/or significant Bonferroni corrected p-values.
