# Supplementary material for: The Social Insurance Literacy Questionnaire (SILQ): Development and Psychometric Evaluation
Source: J Occup Rehabil. 2023 Dec 30;34(3):693–706. doi: 10.1007/s10926-023-10159-7 (PMC11364705; doi:10.1007/s10926-023-10159-7)
Supplement: Supplementary file 4 — Supplementary material 4 (DOCX 121 kb) [file 10926_2023_10159_MOESM4_ESM.docx]

**Supplement 4:** Items that demonstrated DIF for sex or education.
